# Supplementary material for: DMRforPairs: identifying Differentially Methylated Regions between unique samples using array based methylation profiles
Source: BMC Bioinformatics. 2014 May 15;15:141. doi: 10.1186/1471-2105-15-141 (PMC4046028; doi:10.1186/1471-2105-15-141)
Supplement: Additional file 2 — DMRforPairs output for the comparison of A431-MCF7 and NA17018-NA17105. Please start from the HTML files in each folder. Available via the BMC Bioinformatics website. [file 1471-2105-15-141-S2.zip › 1394847754114233_MOESM2_ESM/A431_MCF7/figures/36939.pdf]

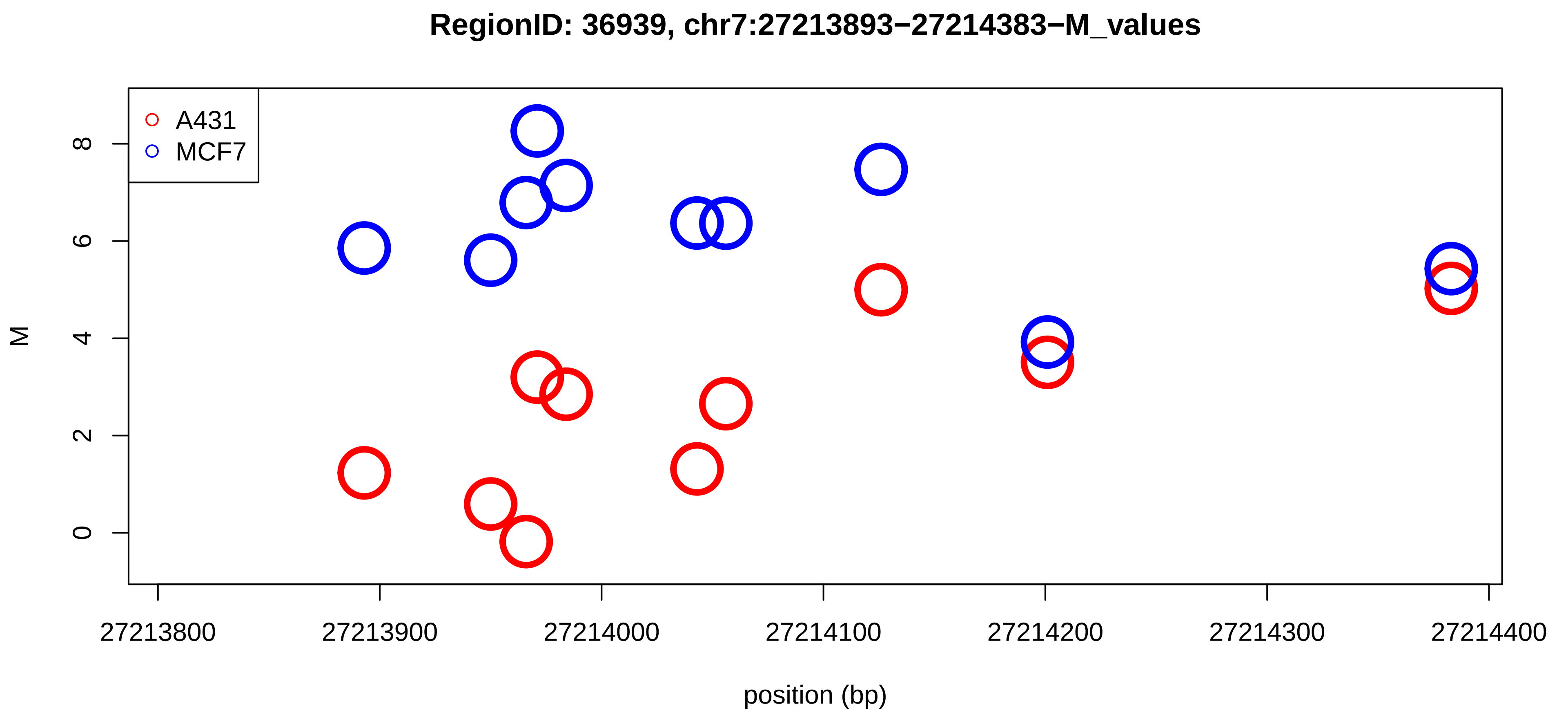
RegionID: 36939, chr7:27213893−27214383−M_values
A431
MCF7
M
0
2
4
6
8
27213800
27213900
27214000
27214100
27214200
27214300
27214400
position (bp)

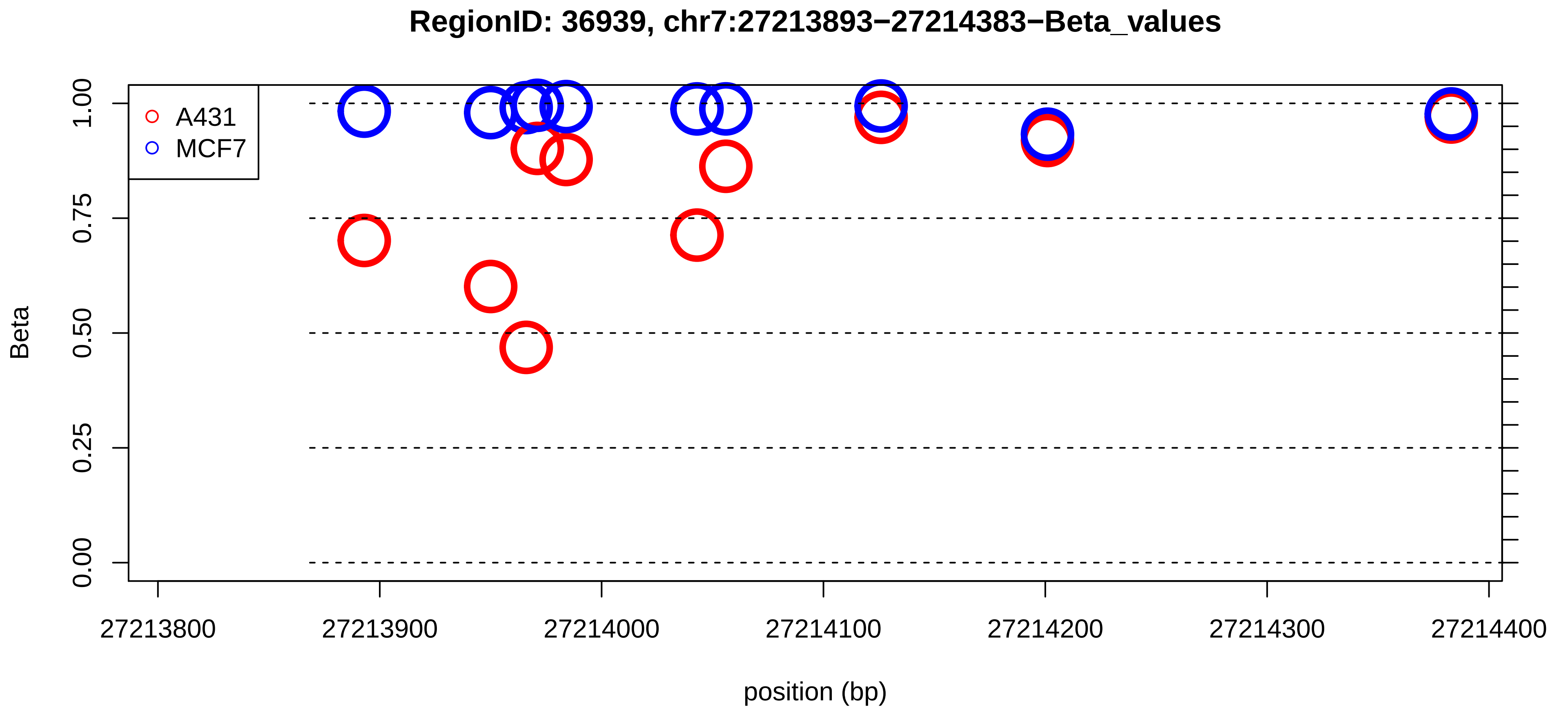
RegionID: 36939, chr7:27213893−27214383−Beta_values
A431
MCF7
Beta
0.00
0.25
0.50
0.75
1.00
27213800
27213900
27214000
27214100
27214200
27214300
27214400
position (bp)

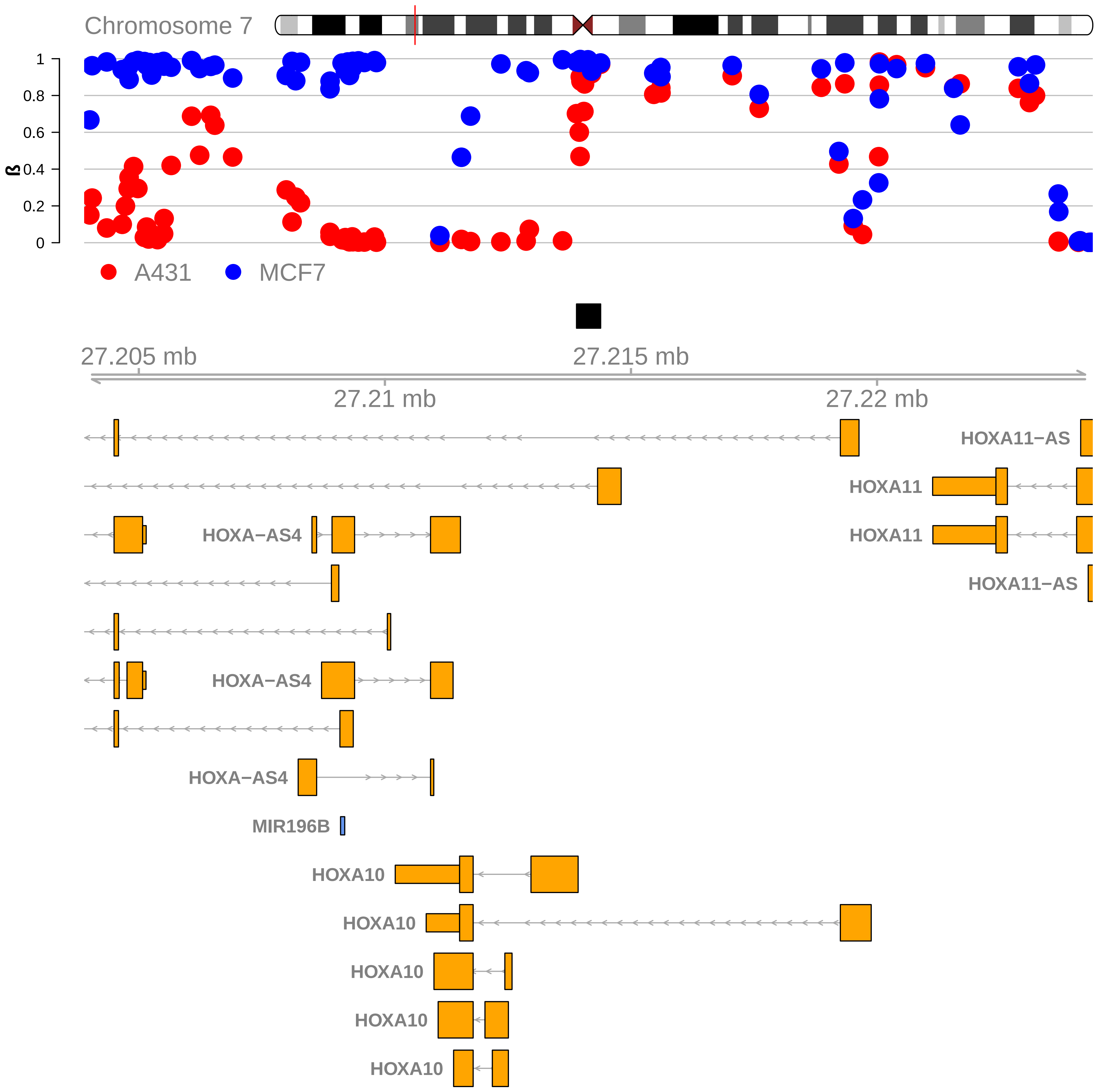

Chromosome 7
1
0.8
0.6
ß 0.4
0.2
0
A431
MCF7
27.205 mb
27.215 mb
27.21 mb
27.22 mb
HOXA11−AS
HOXA11
HOXA−AS4
HOXA11
HOXA11−AS
HOXA−AS4
HOXA−AS4
MIR196B
HOXA10
HOXA10
HOXA10
HOXA10
HOXA10
